# Blood pressure control in hypertensive patients attending a rural community health centre in Gauteng Province, South Africa: A cross-sectional study

**DOI:** 10.4102/safp.v64i1.5403

**Published:** 2022-03-28

**Authors:** Sergius C. Onwukwe, Nnabuike C. Ngene

**Affiliations:** 1 Department of Public Health, Faculty of Health Science, University of Liverpool, Liverpool, United Kingdom; 2 Department of Family Medicine, Faculty of Health Sciences, University of the Witwatersrand, Johannesburg, South Africa; 3 Department of Obstetrics and Gynaecology, Faculty of Health Sciences, University of the Witwatersrand, Johannesburg, South Africa; 4 Department of Obstetrics and Gynaecology, Leratong Hospital, Krugersdorp, South Africa

**Keywords:** blood pressure control, adherence to treatment, diabetes, hypertension, obesity

## Abstract

**Background:**

Hypertension is a major cause of morbidity and mortality and its control has important clinical and socio-economic benefits to the family and community. Unfortunately, the extent of blood pressure (BP) control and its potential predictors in hypertensive patients in many rural communities in low-resource settings are largely unknown. This study assessed the extent of uncontrolled BP and its predictors amongst hypertensive patients accessing primary health care in a rural community in South Africa.

**Methods:**

This cross-sectional study included 422 randomly selected hypertensive patients. Demographic and clinical data were collected using structured face-to-face questionnaire supplemented by respondents’ clinical records.

**Results:**

Obesity plus overweight (*n* = 286, 67.8%) and diabetes (*n* = 228, 54.0%) were the most common comorbidities. Treatment adherence was achieved in only 36.3% and BP was controlled to target in 50.2% of the respondents. Significant predictors of uncontrolled BP were poor treatment adherence (odds ratio [OR] = 15.88, 95% confidence interval [CI] = 8.96, 28.14, *p* < 0.001), obesity compared with normal weight and overweight (OR = 3.75, 95% CI = 2.17, 6.46, *p* < 0.001) and being a diabetic (OR = 2.83, 95% CI = 1.74, 4.61, *p* < 0.001).

**Conclusion:**

Poor adherence to treatment was the major predictor of uncontrolled BP. The increase in uncontrolled BP in the presence of diabetes and/or obesity as risk predictors, indicates the need for appropriate behaviour change/interventions and management of these conditions in line with the health belief model (HBM). We also propose the use of Community-Based Physical and Electronic Reminding and Tracking System (CB-PERTS) to address poor treatment adherence.

## Introduction

Hypertension and its control have important clinical, public health and socio-economic implications for healthcare systems, particularly in low- and middle-income countries where there are reports on increased morbidity and mortality from non-communicable diseases.^1,2^ Globally, hypertension is a major cause of mortality, accounting for 7.0–9.4 million deaths annually, and affects approximately 1 billion people, this being projected to increase by over 500 million by 2025.^1,3,4,5,6^ Currently, the global prevalence of hypertension is estimated to be 59% (55% – 62%) in women, and 49% (46% – 52%) in men, and these are close to the levels reported in South Africa.^5,6^ Hypertension is ranked amongst the top seven causes of death in South Africa, the Black African population being the most affected.^7^ These hypertension-related deaths are mainly because of cardiovascular complications of uncontrolled blood pressure (BP), such as heart failure, ischaemic heart disease (IHD), stroke and kidney failure,^2,8^ these being amongst the most common clinical, public health and socio-economic challenges of uncontrolled BP.

There are different types of antihypertensive medications that are used to treat hypertension in the public sector in South Africa: hydrochlorothiazide – a thiazide diuretic; amlodipine – a calcium channel blocker (CCB), and enalapril – an angiotensin converting enzyme inhibitor (ACE-I) are the most common first-line medications that are used in the treatment of hypertensive patients in primary health care (PHC) settings.^9,10^ These medications are used either as a monotherapy or in different combinations, depending on the presence or absence of compelling clinical indications.^10^ Despite the widespread availability of these antihypertensive medications, controlling BP to recommended targets remains unsatisfactory, especially in poorly resourced countries that are faced with various health system challenges^11^ evident in South Africa^7,9,12^ and most other sub-Saharan African countries.^13,14^ Challenges related to hypertensive patients’ health behaviour such as poor motivations to commit to treatment, lack of adherence to treatment guidelines by clinicians and malfunctioning/underfunding of the healthcare system have been associated with poorly controlled BP.^15,16^

On account of the foregoing discussion, it is important to emphasise that the extent of poorly controlled BP in many rural communities in South Africa and its associations with socio-demographic and clinical factors are largely unknown.^7,9,12^ Thus, in line with the health belief model (HBM), it is envisaged that the findings of this study would be used to design intervention programmes to improve the control of BP, prevent the complications of hypertension, improve the patients’ quality of life, and reduce the cost burden associated with treatment to the patients and the healthcare system.^17,18^ Therefore, this study is aimed to determine the extent to which BP was controlled amongst hypertensive patients accessing care at a large rural PHC centre in Sedibeng District, South of Johannesburg, South Africa and to establish the factors associated with uncontrolled BP.

## Materials and methods

### Study design, setting and period

This was a cross-sectional study undertaken in the day clinic of Levai Mbatha Community Health Centre (CHC), the largest PHC clinic in Sedibeng District, South of Johannesburg, South Africa. This clinic serves a predominantly rural population of over 132 000 inhabitants and accepts patient referrals from 10 PHC clinics in the district for various primary health care services. Data collection was from November 2015 to January 2016.

### Study population, sample size and sampling methods

All hypertensive patients 18 years and above, accessing care at the study setting, were eligible participants. Hypertension was defined as systolic BP ≥ 140 and diastolic BP ≥ 90 mmHg following repeated measurements.^19^ All newly diagnosed hypertensive patients with no previous follow up, too ill to answer questions or needed urgent treatment were excluded. According to the clinic records, the estimated number of hypertensive patients seen yearly is approximately 12 000. The minimum required sample size (*n*) was estimated using the following equation:^17^


n=Z2P(1−P)/e2 [Eqn 1]

where *Z* is the critical value for a 95% confidence level, e is the considered acceptable margin of error of 5% and P is the assumed proportion of 50% of those with poorly controlled BP in the population of patients with 422 eligible participants who were systematically selected at random.^20^ Thus, on each day of the data collection, every eligible third hypertensive patient was recruited.

### Data collection

The survey instrument was a validated questionnaire that had been used previously^9,21^ but with some modifications to the specific context of the setting and to supplement the respondents’ clinical notes in line with the study objectives. Data on demography (age, race, sex, level of education, occupation), lifestyle choices (exercise, alcohol intake, smoking/tobacco use), duration of treatment and adherence (Hill-Bone compliance scale)^21^ were collected.

Data were collected from Patients’ clinical notes on body mass index (BMI; weight in kg divided by height in m^2^), BMI category (defined as: underweight – BMI < 18.5 kg/m^2^, normal weight – 18.5 kg/m^2^ – 24.9 kg/m^2^, Overweight – BMI 25 kg/m^2^ – 29.9 kg/m^2^, obesity class I – BMI 30 kg/m^2^ – 34.9 kg/m^2^, obesity class II – BMI 35.0 kg/m^2^ – 39.9 kg/m^2^, obesity class III – BMI > 40 kg/m^2^),^22^ comorbid illness (bronchial asthma, cardiac failure, diabetes, dyslipidemia, IHD, renal dysfunction), antihypertensive drugs therapy (types of drugs, number of drugs, combination of drugs) and BP measured in each occasion during at least two different clinic visits. The BP, height and weight were measured during clinic visit on the day of the data collection by the triaging healthcare staff as part of the routine process of care. Specifically, BP was measured using a validated and regularly calibrated automated device, and this is in line with the current South African Hypertension Practice Guidelines.^10^ The measurements were taken from the arm of each study participant at rest using an appropriate size cuff. During the measurement, the patient was seated, his or her shoulders supported on the backrest of the chair, legs not crossed, feet placed on the floor and neither the patient nor the healthcare professional was talking. The average of the BP reading from the previous visit and that obtained on the day of data collection/interview were recorded and used as a measure of BP control. The BP measurement were in line with the existing local and international hypertension protocols and guidelines.^10,19^ The BP control targets were based on both the guidelines of the International Society of Hypertension and the South African Hypertension Society.^10,19^ These target BP in the study setting at the time of the study were < 140/90 mmHg for uncomplicated hypertension and ≤ 130/80 mmHg for those with comorbidity such as diabetes and chronic kidney disease (CKD).^20^ Data on measures of CKD, diabetes and dyslipidaemia, as documented in the patients’ files at the time of initial diagnosis were also extracted. In line with the South African Hypertension Guidelines, CKD was defined as estimated glomerular filtration rate (eGFR)  <  60  mL/min, which was obtained from the laboratory values of creatinine and calculation of the estimated GFR using MDRD formula. Dyslipidaemia was defined as total fasting cholesterol > 5.1 mmol/L.^10^

Data on adherence to treatment were collected through structured interview using the evidence-based Hill-Bone compliance therapy scale.^21^ This non-invasive and cost-effective adherence measuring instrument has shown good validity in primary care amongst black South Africans^23^ and African Americans.^21^ The scale is consistent in assessing patient’s behaviour in three main domains of high BP treatment when used to describe adherence in hypertensive patients: reduced salt intake, making appointment and taking medication. Altogether, there are 14 items or questions in this scale, which are all scored in a 4-point Likert scale. Adherence to treatment means more than 90% of the respondents answering ‘all of the time’ to the question, ‘how often do you make the next appointment before you leave the clinic’ and ‘none of the time’ to at least 10 of the 13 remaining questions on the Hill-Bone compliance scale.

The data collection and interview process did not disrupt the participant’s access to care. This was achieved by ensuring that no interview was done until the patients had been seen by their clinicians and had collected their medications.

### Statistical methods

Data were analysed using IBM Statistical Package for the Social Sciences (SPSS) version 24.0 (IBM, United States). All categorical data were presented as frequencies and percentages. Normality check was performed on continuous variables to identify patterns of data distribution. Normally distributed continuous data were presented as mean and standard deviation (s.d.). Independent variables were initially tested for association with BP control using the Chi-square test. Statistically significant variables found in the bivariate analyses were included stepwise in a multivariate logistic regression analysis to determine the factors that remained associated with control of BP when entered into the model alongside the other variables that were shown to be significant. Statistical significance was set at *p* < 0.05.

## Ethical considerations

Permission to conduct the study was given by the Sedibeng District health services. Ethical approval was granted by the Ethics Committees of the University of the Witwatersrand, South Africa (certificate number: M150859) and University of Liverpool, United Kingdom (certificate number: 7-16-2015-01). Study participants gave informed consent before they were enrolled. All personal patient information was de-identified at source and codes used for all participants, thereby, preserving their anonymity.

## Results

**FIGURE 1:**
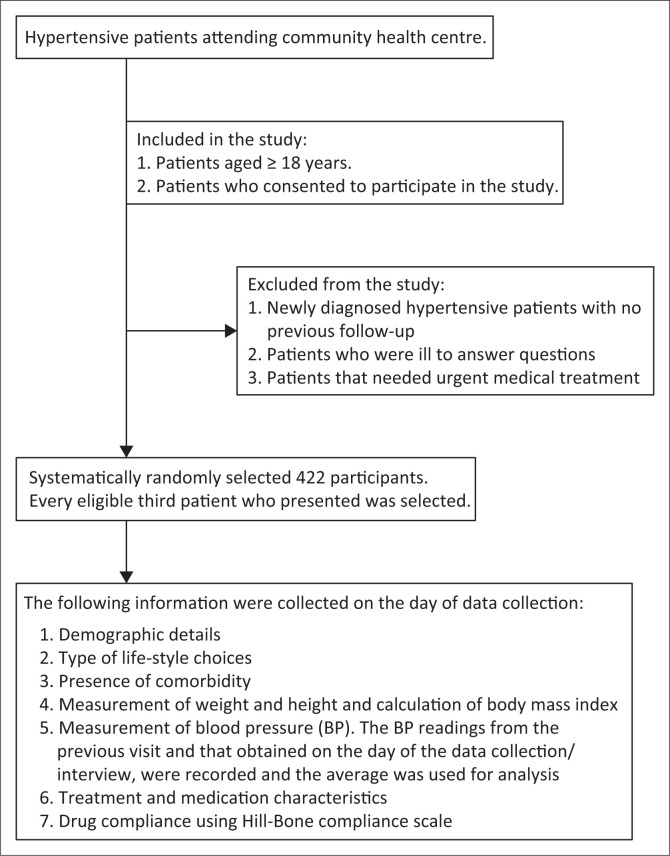
Flow diagram showing study participants and data collection.

Figure 1 is a flow diagram of the recruitment of study participants.

### Demographic details

Of the 422 hypertensive patients’ who participated in the study, all were black people, slightly over half (54.3%, *n* = 229) were men of mean age 59.4 (s.d. = 12.0) years. Whilst women (45.7%, *n* = 193) had a mean age 59.7 (s.d. = 12.3) years. Over half of the participants were unemployed (58.1%, *n* = 245) and the majority had either primary (43.6%, *n* = 184) or secondary school education (40.8%, *n* = 172). The details of the demographic characteristics of the participants are shown in Table 1.

**TABLE 1:** Demographic characteristics of study participants.

Characteristics (*n* = 422)	Frequency	%
**Gender**		
Male	229	54.3
Female	193	45.7
**Age group (years)**		
18–29	1	0.24
30–39	22	5.21
40–49	67	15.9
50 and over	332	78.7
**Race**		
Black people	422	100
White people	0	0
**Occupation**		
Employed	177	41.9
Unemployed	245	58.1
**Education**		
No formal education	49	16.1
Primary school	184	43.6
Secondary school (Matric)	172	40.8
Tertiary	17	4

### Body mass index, comorbidity and blood pressure control level

Table 2 shows the details of the BMI, comorbidity and BP control level of the respondents. Men had a mean BMI of 26.6 (s.d. = 5.1) kg/m^2^, whilst for women it was 30.1 (s.d. = 6.1) kg/m^2^. A total of 54% (*n* = 228) and 50.9% (*n* = 215) had diabetes and dyslipidaemia, respectively. Target BP control, irrespective of comorbidities, was achieved in 50.2% of patients. Mean systolic BP was 138.7 mmHg (s.d. = 15.9) for men and 140.3 mmHg (s.d. = 17.5) for women. Mean diastolic BP was 84.4 mmHg (s.d. = 9.8) for men and 84.8 mmHg (s.d. = 10.9) for women.

**TABLE 2:** Body mass index, comorbidity and blood pressure control.

Characteristics *n* = 422	kg/m^2^	Frequency	%
**BMI category**			
Underweight	< 18.5	6	1.4
Normal weight	18.5–24.9	133	31.5
Overweight	25.0–29.9	148	35.1
Obesity I	30.0–34.9	86	20.4
Obesity II	35.0–39.9	33	7.8
Obesity III	> 40.0	16	3.8
**Comorbid Illness**			
Bronchial asthma	-	49	11.6
Heart failure	-	79	18.7
Diabetes mellitus	-	228	54.0
Dyslipidemia	-	215	50.9
Ischaemic heart disease	-	0	0.0
Renal failure	-	47	11.1
No-comorbidity	-	47	11.1
**Blood pressure control**			
Controlled	-	212	50.2
Uncontrolled	-	210	49.8

BMI, body mass index

### Characteristics of lifestyle choices

Table 3 shows the characteristics of life style choices of the participants. Of those who reported that they did some sort of exercise (*n* = 71, 16.8%), 83.1% did this less than three times a week with an exercise duration of < 30 min in 47 (66.2%). Most of the respondents (*n* = 287, 68.0%) did not drink alcohol, but of those 32.0% (*n* = 135) who did, 104 (77.0%) .0 only occasionally on less than a weekly basis. The majority of participants (*n* = 349, 82.7%) reported that they smoked or used tobacco products.

**TABLE 3:** Characteristics of lifestyle choices.

Characteristics (*n* = 422)	Number	%
**Do you do any type of exercise?**		
Yes	71	16.8
No	351	83.2
**If yes, how many times a week?**		
< 3 times	59	83.1
3–5 times	12	16.9
**What is the duration of each exercise?**		
< 30 min	47	66.2
30–45 min	24	33.8
**Do you drink alcohol?**		
Yes	135	32.0
No	287	68.0
**If yes, how much do you drink in a week?**		
Occasional/not every week	104	77.0
< 3 standard bottles per week	29	21.5
> 3 standard bottles	2	1.5
**Do you smoke or use any type of tobacco?**		
Yes	349	82.7
No	73	17.3

### Treatment/medication characteristics

Table 4 shows the treatment/medication characteristics of respondents. The most widely prescribed antihypertensive medication by the attending clinicians was enalapril (87.9%), amilodipine (71.3%) and hydrochlorothiazide (60.2%). The medications were mainly used in the most appropriate combinations (*n* = 405, 96.0%) with the commonest pattern being three or more (*n* = 285, 67.3%). However, the majority of patients (*n* = 269, 63.7%) were not fully adherent with the hypertensive treatment, with the mean duration of treatment being 10.0 (s.d. = 7.6) years in women and 8.5 (s.d. = 6.8) years in men.

**TABLE 4:** Treatment/medication characteristics of respondents.

Characteristics (*n*) = 422	Frequency	%
**ACE- inhibitor (ACE-I) (Medication type I)**		
Enalapril	371	87.9
Perindopril	1	0.2
**Calcium channel blocker (CCB) (Medication type II)**		
Amlodipine	301	71.3
Nifedipine	3	0.7
**Diuretics (Medication type III)**		
Furosemide	86	20.4
Spironolactone	4	0.9
Hydrochlorothiazide	254	60.2
**Beta-blocker (BB)**		
Atenolol	50	11.8
Carvedilol	4	0.9
**Number of drugs taken**		
One	19	4.5
Two	118	28.0
Three or more	285	67.5
**Combination of drugs**		
Correct	405	96.0
Incorrect	17	4.0
**Compliance/adherence to treatment**		
Compliant	153	36.3
Non-compliant	269	63.7

### Responses to the Hill-Bone compliance (adherence) scale

Table 1-A1 shows the questions and responses to the Hill-Bone compliance scale.^21^ In this table, the reply of ‘none of the time’ to 13 of the 14 items on this scale was achieved by over 90% of the respondents in only six of the 13 items or questions in the scale, signifying poor adherence. However, adherence to follow up appointments was good because almost all the respondents (97.5%) answered ‘all of the time’ to the remaining one question on the scale: ‘how often do you make the next appointment before you leave the clinic’.

### Determinants of blood pressure control

Preliminary analysis using the Chi-square test indicated that uncontrolled BP was significantly associated with two demographic factors: the sex of the patient (*p* = 0.019) and their BMI category (*p* < 0.001). Uncontrolled BP was also associated with the presence of certain comorbid conditions: bronchial asthma (*p* = 0.011), diabetes (*p* < 0.001) and CKD (*p* = 0.041). In addition, the number of drugs prescribed (*p* = 0.01), the correct combination of drugs (*p* = 0.025) and adherence to treatment (*p* < 0.001) were found to be associated with uncontrolled BP.

These factors were therefore included in the multivariate logistic regression analysis to identify which ones were most likely to indicate possible uncontrolled BP. Body mass index, diabetes and treatment adherence remained in the model following stepwise elimination of those factors that were not significant in the model. The odds ratios (ORs) of obesity when compared with normal weight and overweight independently were not significantly different, and the BMI category was reconsidered as a dichotomous variable normal weight and overweight as compared with obesity. The multivariate logistic regression analysis showed the association between the determinant factors and uncontrolled BP (Table 1-A2). Although the BMI category of obesity compared with normal and overweight (OR = 3.74, *p* < 0.001) and the presence of diabetes (OR = 2.83, *p* < 0.001) clearly contribute to the association with uncontrolled BP, the major factor was the lack of adherence to treatment (OR = 15.88, *p* < 0.001).

## Discussion

This study found that BP was controlled to target in 50.2% of the hypertensive patients, all of whom were black people and accessing PHC. Our finding is in line with the BP control report by Berry et al.^24^ in one of the first nationally representatives estimates of the burden of hypertension and extent of hypertensive care in South Africa. Similar to what we found, almost half of the treated patients are not controlled.^24^ It is important to emphasise that although most studies of BP control in South Africa have reported lower control rates,^7,12^ with the exception of a 57.0 % BP control rate reported by Onwukwe and Omole,^9^ we are of the opinion that our control rate is still unsatisfactory. This is because of the fact that almost half of the respondents whose BP was not controlled to recommended levels could be predisposed to various cardiovascular complications and these have important public health implications such as stroke, congestive heart failure, peripheral vascular disease and CKD.^2,8^ This lack of BP control occurred despite the observation that the prescribed antihypertensive medications combinations were appropriate in most patients (96%) and in line with the existing treatment guidelines and consistent with previous studies.^9,12^

In this study notably, the participants were essentially black patients. The frequency of use of the first-line antihypertensive drugs were enalapril, an ACE-I (87.9%); amlodipine, a CCB (71.3%), and hydrochlorothiazide, a thiazide diuretics (60.2%). We found that these drugs were used in appropriate combinations in 96% of the patients and appropriate drug combination is recommended in the current South African hypertension treatment and other international guidelines.^10,19^ Specifically, the Creole Clinical Trial that was performed on black patients in sub-Saharan Africa emphasises the compelling need to use appropriate combination therapy to treat hypertension.^25^ In that study, amlodipine combined with hydrochlorothiazide or perindopril was found to be more efficacious than perindopril combined with hydrochlorothiazide in reducing BP to optimal levels.^25^ In African Americans, achieving optimal BP control goals with these antihypertensive drugs combinations is very hard according to the evidence from the Reasons for Geographic and Racial Differences in Stroke (REGARDS) study.^26,27^ This is because of a combination of factors such as dietary pattern, predisposition to increased salt sensitivity and treatment resistant hypertension, which effectively raises their BP medication requirements resulting in suboptimal control of the BP.^26,27^ In those with resistant hypertension, the use of spironolactone have been found to improve BP control, and when a thiazide or thiazide such as diuretic is indicated, chlorthalidone or indapamide have been shown to be more efficacious than hydrochlorothiazide in lowering BP.^28^ A total of 84% of the patients in this study had comorbid diabetes, heart failure and CKD either alone or in combination, thus, requiring a compelling use of an ACE-I for effective and optimum treatment.^10^ Apart from its recommended use in the control of BP preferably in combination with amlodipine in uncomplicated hypertension, the compelling rationale for the use of ACE-I is to slow or halt the progression of microvascular complications associated with diabetes, heart failure or CKD.^29^

Optimisation of prescribed antihypertensive medication is currently supported by evidence because patients who are not receiving the correct dose and combination of their medications finds it difficult to achieve optimal BP control.^10,19^ In this study, we do not have full data on the optimisation of doses of the antihypertensive medications used by the patients or the actual reasoning for the preference to use a particular antihypertensive drug.

Risk perception to uncontrolled BP and poor adherence to recommended treatment for hypertension may be a health behavioural issue, which is well rooted in the HBM and can be used to design interventions to address it.^17,18^ Specifically, in terms of behaviour, the HBM is a health behaviour theoretical framework that can be used to explain why people take actions to control their illness such as hypertension and to suggest/design interventions against it.^17,18^ In practice, the constructs of the HBM are perceived susceptibility, severity, benefits, barriers, cues to actions and self-efficacy.^17^ Although this study was not designed to use these constructs to predict BP control, however, it is assumed that it can be used to design interventions against the predictors of BP control emanating from the study.^17,18^ This is because evidence from the use of the HBM as a behavioural change framework for disease conditions indicates that people’s beliefs about whether or not they are susceptible to a disease such as hypertension and their perceptions of the benefits of trying to avoid it, influences their readiness to act, which includes accepting interventions directed at risk reductions for the disease.^17,18^

In our study, only 36.3% of the respondents were fully adherent to hypertensive treatment and this is consistent with previous reports.^13,30^ The subsequent observation that lack of adherence to treatment is the most significant factor associated with uncontrolled BP (OR = 15.88, *p* < 0.001) also confirms previous findings.^31^ Factors affecting adherence to treatment includes pill burden (resulting from lack of use of fixed or single dose pill combinations) and medication side effects especially cough and angioedema when an ACE-I such as enalapril is used.^10,31,32,33^ Specifically, in this study, we did not directly assess the side effect of drugs but have a direct and quantifiable data on pill burden because almost 70% of the patients were prescribed three or more different antihypertensive pills that were not in fixed dose combinations: a major failure in the public health sector in South Africa. It is important to emphasise that in line with other international treatment guidelines, current local guidelines on treatment of hypertension recommends the use of fixed or single dose pills to help patients deal with the issue of poor medication adherence and control of BP.^10,19^ Unfortunately, fixed dose combination of antihypertensive pills are presently not available in the South African Public health sector, therefore, patients are made to depend on multiple pills, which potentially predisposes them to poor adherence. Furthermore, in our study, as previously expressed, we assessed adherence including its underlying contributors such as medication side effects (indirect assessment) and pill burden (direct assessment) by the use of the 14-item or questions in the validated Hill-Bone compliance (adherence) scale.^21^ This adherence scale is relatively subjective, non-invasive and cost-effective tool that also has its limitations.^21^ However, it is important to emphasise that the difficulty in accurately assessing adherence to hypertensive treatment is well-documented in literature and this has led to the suggestion for the use of combination of methods.^33^ Based on this, direct measurement of ingested antihypertensive drugs in blood or urine as performed in a study in the United Kingdom and Czech Republic appears to be the best method to accurately assess and document adherence and its effect on BP control.^34^ Unfortunately, because of economic cost and invasive nature of this biochemical method of assessing adherence, we were unable to fit it into our study methods.^33^ Furthermore, given that the relatively subjective method that we used appears to be less accurate compared with the biochemical method, our reported level of adherence in relation to BP control needs to be interpreted with caution. This becomes more imperative as many of the questions in the 14-item Hill-Bone compliance scale actually assesses hypertension treatment behaviour rather than adherence based on actual ingestion of the antihypertensive drug.^21^ This may be an indication of why our reported level of adherence to treatment is very poor whilst actual BP control reached a modest 50.2% threshold. However, as already indicated, our reported BP control rate is in line with a previous finding in South Africa.^24^

Comorbid obesity and diabetes observed in this study, appear to be common in South Africa and tend to be important factors for cardiovascular disease.^36^ In line with previous studies, both are associated with prolonged poor BP control^4,7,8^ and in practice has socio-economic consequences of increasing the healthcare costs of treatment and productive workforce disability.^16,35^ Specifically, we found that obesity compared with normal weight and overweight was a strong predictor (OR = 3.75, *p* < 0.001) of uncontrolled BP, which is consistent with observations from previous studies.^37^ Also, consistent with the findings of Reboldi et al.,^38^ 54% of the patients in this study had comorbid diabetes. This was also shown to be a risk factor for uncontrolled BP (OR = 2.83, *p* < 0.001) and is similar to the reports by Duggirala et al.^39^ and Morgado et al.^31^ Diabetes is a regularly implicated risk factor for nephropathy, retinopathy and neuropathy,^39^ indicating the need for regular screening for these conditions in primary care. Also, approximately 10% of patients in our study had comorbid renal disease, which was not associated with uncontrolled BP. However, this finding needs to be interpreted with caution as a previous study revealed that silent renal disease not usually diagnosed/identified at screening was present in 25% of hypertensive men and 6% of women.^40^ This calls for concern, especially, if the number of patients with renal disease (CKD) in this study is extrapolated to a population level as there are usually large costs and other public health consequences associated with managing these patients.

There is overwhelming evidence showing that lifestyle choices associated with inadequate physical activity, habitual smoking and excessive alcohol intake (e.g. poor diet, excessive salt intake), are major public health concerns because of their associations with raised BP and its exacerbations.^41,42^ In this study, it was observed that a few participants (16.8%) were meeting exercise goals and alcohol intake was extremely low (32%). Surprisingly, we found that 82.5% either smoke or use tobacco products, which is a leading risk factor for cardiovascular events including hypertension. However, it is important to note that the link between smoking/use of tobacco and BP is debatable and according to the recommendations of the South African Hypertension guideline, in people who are already hypertensive, cessation of use of tobacco products does not readily translate to a drop in systolic BP.^10^ This is in contrast to other life style modifications such as moderate weight loss, use of Dietary Approaches to Stop Hypertension (DASH) diet, reduction in salt/sodium use, physical activity and moderation of alcohol intake, which result in varying degrees of reduction in systolic BP in people who are already hypertensive.^10^ On the strength of our findings, the historical role that lifestyle modifications play in the adjunctive management of cardiovascular diseases including hypertension needs to be re-enforced during patients’ encounters with attending PHC clinicians, and the HBM may be useful in this circumstance.^17,18^

### Strengths and limitations

Our findings may not be generally applicable to other racial groups in South Africa, as only black patients were studied and there may be peculiar demographic and clinical differences in BP control that would need to be tested and compared with other racial groups.^15^ The interview aspect of the study including the responses to the items in the Hill-Bone compliance (adherence) scale was mainly based on self-report, which may not only create a form of information bias but being a cross-sectional design, cannot infer causation.^43^ This may be a plausible explanation for the disparity between the level of BP control and treatment adherence. However, the use of evidence-based BP control targets and measurements taken in the clinic from patients, and the combination of face-to-face interview and patients’ clinical records for data collection, may have helped to ensure greater certainty in these aspects of the data.

### Recommendations on future study on treatment adherence

We align with the recommendations of the South African Hypertension treatment guidelines for the use of fixed dose or single pill combinations to reduce pill burden in patients.^10^ In addition, apart from the constructs of the HBM that can be used to screen for risk perceptions for hypertension and barriers to treatment adherence, which is necessary for the design of interventions, an additional measure to prevent poor adherence to treatment in patients with chronic conditions such as hypertension is Community-Based Physical and Electronic Reminding and Tracking System (CB-PERTS). This is for use in patients identified in the clinic to have poor treatment adherence. The components of CB-PERTS in authors’ view will include: (1) scheduled home visits by community healthcare workers to encourage adherence, (2) supportive interview and (3) establishing an electronic alert system such as mobile phones that may be used by a patient to notify the healthcare professional about any difficulty with treatment adherence or used by healthcare professional to remind a patient about the need for treatment adherence (thus, the electronic system is a two-way patient-healthcare professional alert system). Incidentally, our proposal on the use of digital system to assist in managing chronic hypertension is supported by WHO recommendation.^44^ We recognise that a previous study (STAR) in South Africa that used the digital system (mobile-phone short message system) strategy produced minimal changes in adherence.^45^ In contrast, our proposal is a combination of three strategies, which include the use of the mobile-phone digital system, and we are of the view that it may produce a different result if it is given an opportunity for a trial in primary care. Additional study is underway to implement/assess the effectiveness of the CB-PERTS in improving treatment adherence.

## Conclusion

This study, which used a combination of interview supplemented by clinical records, found that BP was controlled to target in approximately half of the hypertensive patients. Adherence to treatment may be the most important objective in the PHC setting for hypertensive patients. Whilst it is known that the HBM can be used to design interventions against observed predictors of uncontrolled BP including poor adherence to treatment, CB-PERTS as proposed in this report should be tested and brought to use if found effective in improving treatment adherence. Hypertensive patients with diabetes and or obesity require special consideration, as these conditions appear to be an independent risk factor for uncontrolled BP. Therefore, there is a need for regular screening, health education and promotion initiatives including behavioural change interventions aimed at the identified risk factors and predictors of uncontrolled BP in primary care, and this is in line with the health belief model.

## Appendix 1

**TABLE 1-A1:** Questions and responses to the Hill-Bone compliance (adherence) scale.

Questions: Characteristics (*n* = 422)	Responses (%)			
	None of the time	Some of the time	Most of time	All of the time
How often do you forget to take your high BP medication?	66.6	32.2	1.2	-
How often do you decide not to take your high BP medication?	92.6	7.4	-	-
How often do you eat salty food?	46.4	49.8	3.8	-
How often do you shake salt on your food before you eat?	79.7	14.9	5.4	-
How often do you eat fast food?	47.5	32.9	17.5	2.1
How often do you make the next appointment before you leave the clinic?	1.4	0.4	0.7	97.5
How often do you miss scheduled appointment?	89.0	11.0	-	-
How often do you forget to get your prescriptions filled?	90.9	9.1	-	-
How often do you run out of high BP pills?	85.7	14.3	-	-
How often do you skip your high BP medicine before you go to the clinic?	55.9	43.4	0.7	-
How often do you miss taking your high BP pills when feeling better?	95.0	5.0	-	-
How often do you miss taking your high BP pills when you feel sick?	97.0	3.0	-	-
How often do you take someone else’s high BP pills?	94.3	5.7	-	-
How often do you miss taking your high BP pills when you are careless?	91.0	9.0	-	-

*Source*: Kim MT, Hill MN, Bone LR, et al. Development and testing of the Hill-Bone compliance to high blood pressure therapy scale. Prog Cardiovas Nurs. 2000;15(3):90–96. https://doi.org/10.1111/j.1751-7117.2000.tb00211.x. Reproduced with permission from John Wiley and Sons.

BP, blood pressure

**TABLE 2-A1:** Multivariate logistic regression analysis for factors associated with uncontrolled blood pressure.

Predictor	Β	SEβ	Wald’s ϗ	*df*	*p*-value	OR	95% CI
Constant	−2.269	0.228	62.059	1	< 0.001	NA	-
Obesity	1.320	0.278	22.603	1	< 0.001	3.745	2.173,6.455
Diabetes	1.040	0.249	17.436	1	< 0.001	2.830	1.737,4.611
Compliance	2.765	0.292	89.736	1	< 0.001	15.879	8.961,28.137
Model Likelihood ratio test			179.600	3	< 0.001	-	-
**Likelihood ratio test of reduced model**							
Obesity	-	-	24.720	1	< 0.001	-	-
Diabetes	-	-	18.090	1	< 0.001	-	-
Compliance	-	-	122.550	1	< 0.001	-	-

Note: Cox and Snell *R*^2^ = 0.347. Nagelkerke *R*^2^ = 0.462. McFadden = 0.307. IBM SPSS statistics, Version 24 was used.

OR, odds ratio; CI, confidence interval; SE, standard error; *df*, degree of freedom; NA, not applicable.
